# A 3D-CNN with temporal-attention block to predict the recurrence of atrial fibrillation based on body-surface potential mapping signals

**DOI:** 10.3389/fphys.2022.1030307

**Published:** 2022-11-08

**Authors:** Gaoyan Zhong, Xujian Feng, Han Yuan, Cuiwei Yang

**Affiliations:** ^1^ The Center for Biomedical Engineering, School of Information Science and Technology, Fudan University, Shanghai, China; ^2^ Key Laboratory of Medical Imaging Computing and Computer Assisted Intervention of Shanghai, Fudan University, Shanghai, China

**Keywords:** atrial fibrillation recurrence, attention, body surface potential mapping, 3D convolutional neural network (3D CNN), isopotential map

## Abstract

Catheter ablation has become an important treatment for atrial fibrillation (AF), but its recurrence rate is still high. The aim of this study was to predict AF recurrence using a three-dimensional (3D) network model based on body-surface potential mapping signals (BSPMs). BSPMs were recorded with a 128-lead vest in 14 persistent AF patients before undergoing catheter ablation (Maze-IV). The torso geometry was acquired and meshed by point cloud technology, and the BSPM was interpolated into the torso geometry by the inverse distance weighted (IDW) method to generate the isopotential map. Experiments show that the isopotential map of BSPMs can reflect the propagation of the electrical wavefronts. The 3D isopotential sequence map was established by combining the spatial–temporal information of the isopotential map; a 3D convolutional neural network (3D-CNN) model with temporal attention was established to predict AF recurrence. Our study proposes a novel attention block that focuses the characteristics of atrial activations to improve sampling accuracy. In our experiment, accuracy (ACC) in the intra-patient evaluation for predicting the recurrence of AF was 99.38%. In the inter-patient evaluation, ACC of 3D-CNN was 81.48%, and the area under the curve (AUC) was 0.88. It can be concluded that the dynamic rendering of multiple isopotential maps can not only comprehensively display the conduction of cardiac electrical activity on the body surface but also successfully predict the recurrence of AF after CA by using 3D isopotential sequence maps.

## 1 Introduction

Atrial fibrillation (AF) is the most common cardiac arrhythmia with a prevalence of 10%–18% in people aged over 80 (Zoni-Berisso et al., 2014). Although catheter ablation (CA) therapy can effectively treat AF, the recurrence rate of AF is still high, and the mechanism of recurrence is not clear (Schotten et al., 2011; Calvo et al., 2018; McCann et al., 2021). At present, predicting postoperative recurrence in AF patients based on preoperative clinical baseline data would enable the selection of the best personalized treatment for AF patients.

Various body surface electrocardiogram (ECG) and intracardiac electrogram (EGM) predictors associated with AF recurrence after CA have been reported. Everett et al. (2001) concluded that the spectrum of AF signals contains information related to its tissue and can be used to predict the successful termination of AF in ten dogs. Takahashi et al. (2006) found that a higher organization index (OI) of atrial EGM was associated with the termination of AF during limited ablation; this parameter may be useful for anticipating the extent of ablation. Meo et al. (2013) argued that the amplitude variability of AF waves (f-waves) could be characterized by multi-lead ECG to predict the prognosis of CA. Szilágyi et al. (2018) used body-surface ECG and intracardiac EGM signals for spectrum analysis and found that dominant frequency (DF), regularity index (RI), and OI could be used to predict AF recurrence. Furthermore, most of the methods based on ECG complexity investigated to date have been determined both in the frequency (Alcaraz et al., 2016; Hidalgo-Muñoz et al., 2017) and time domain (Nault et al., 2009) or in AF cycle length (Matsuo et al., 2009), and a few by sample entropy (Alcaraz et al., 2011). Nevertheless, the acquisition of EGM is difficult for its trauma, and some body-surface ECG, like the standard 12-lead ECG or single-lead ECG, could not provide sufficient spatial–temporal information on atrial activity to predict AF recurrence.

Body-surface potential mapping signals (BSPM) can not only provide sufficient body surface information but also effectively characterize the atrial complexity of patients with AF. Bonizzi et al. (2010) demonstrated that BSPMs outperform standard single-lead analysis and proposed a novel automated approach to quantitatively assess the degree of the spatial–temporal organization of atrial activity (AA) during AF. Zhang et al. (2018) suggested that the fast Fourier transform (FFT) algorithm is a useful and convenient way to evaluate the rhythm of BSPMs in AF patients, which is important for identifying some hypotheses to predict the recurrence of AF. Their study also demonstrated that multi-channel mapping is superior to standard 12-lead ECG. Meo et al. (2018) proposed a marker from BSPMs to quantify AF complexity that could be used to select patients eligible for AF ablation. Marques et al. (2020) used frequency and phase analyses of BSPM maps to reveal distinct behavior between arrhythmias. Li et al. (2018) proposed a deep learning algorithm based on BSPMs to predict AF recurrence after CA. However, most studies quantify AF complexity using traditional machine-learning methods, and few studies use deep learning to predict AF recurrence after CA based on the three-dimensional (3D) spatial–temporal features of BSPMs.

Due to the volume of BSPMs and the difficulty of distinguishing and quantifying important features, electrical image sequence representation is a common visualization tool in evaluating and understanding BSPMs (Brook and MacLeod, 1997). Common methods include isochrone maps, isopotential maps, integral maps, isoarea or isointegral maps, and phase maps (Brook and MacLeod, 1997; Rogers et al., 1998). Isopotential maps are obtained by directly plotting the mapped ECG data—the voltage amplitude—on the model without modification. This drawing will not add any additional information nor any data processing, so it will not lose any mapping information.

In this study, 3D visualization techniques were used to deeply explore the temporal evolution of BSPMs to predict the recurrence of AF. It takes a step from previous research and proposes a noninvasive isopotential map-based approach for the evaluation of AF complexity. We here propose a new method for extracting the spatial–temporal characteristics of cardiac activations during AF and realize the prediction of AF recurrence by inputting 3D isopotential sequence maps into a 3D convolutional neural network (3D-CNN). This method not only provides the overall propagation pattern of ECG signals on the body surface but also successfully predicts the recurrence of AF. At the same time, the innovative temporal-attention block solves the problem of the 3D input signal not being able to effectively extract important information based on time series.

## 2 Material and methods

### 2.1 Data collection

BSPM data from 33 patients with clinical AF were collected before and after macrovascular surgery at West China Hospital of Sichuan University; 14 AF patients with radiofrequency surgery ablations and successful electrical cardioversion within 3–4 weeks had been the subject of continuous follow-up studies for 1 year. The study was approved by the ethics review board of West China Hospital, Sichuan University, and written informed consent was obtained from all patients upon admission. Moreover, their personal information was anonymized and de-identified prior to analysis. Table 1 lists their clinical characteristics and the basic information.

**TABLE 1:** Fundamental information and clinical characteristics of subjects.

ID	Age	Sex	Height	Weight	Preoperative rhythm	Description	Surgical process and treatment plan	Recurrence	Segments
13	53	Male	170	66	AF	RHD^a^: MS^b^, MR^c^, TR^d^	MVR^g^, TVP^h^, Maze^j^	No	72
14	52	Male	—	—	AF	RHD^a^: MS^b^, MR^c^, TR^d^	MVR^g^, Maze^j^	No	106
16	50	Male	164	58	AF	RHD^a^: MS^b^, AS^f^, AR^e^	MVR^g^, Maze^j^	No	86
17	50	Male	170	68	AF	RHD^a^: MS^b^, MR^c^, AR^e^	MVR^g^, Maze^j^	No	82
18	69	Male	173	56	AF	RHD^a^: MS^b^, TR^d^	MVR, Maze^j^	Yes	69
19	46	Female	156	55	AF	RHD^a^: MS^b^, MR^c^, TR^d^	MVR^g^, TVR^h^, Maze^j^	Yes	62
20	44	Female	155	55	AF	RHD^a^: MS^b^, MR^c^, TR^d^	MVR^g^, TVR^h^, Maze^j^	No	68
21	50	Female	155	45	AF	RHD^a^: MS^b^, TR^d^	MVR^g^, TVR^h^, Maze^j^	No	74
22	46	Male	173	67	AF	RHD^a^: MS^b^, TR^d^	MVR^g^, TVR^h^, Maze^j^	No	91
23	65	Female	156	57	AF	RHD^a^, MS^b^, MR^c^, TR^d^	MVR^g^, TVR^h^, Maze^j^	No	70
24	42	Female	157	80	AF	RHD^a^: MR^c^, TR^d^	MVR^g^, TVR^h^, Maze^j^	Yes	108
25	62	Female	152	55	AF	RHD^a^: MS^b^, AR^e^	MVR^g^, TVP^h^, Maze^j^	No	88
26	43	Female	153	49	AF	RHD^a^: MS^b^, TR^d^	MVR^g^, AVR^i^, TVR^h^, Maze^j^	No	76
30	50	Female	154	47	AF	RHD^a^: MS^b^, MR^c^, TR^d^	MVR^g^, TVR^h^, Maze^j^	Yes	120

^a^ RHD, rheumatic heart disease.

^b^ MS, mitral valve stenosis.

^c^ MR, mitral valve regurgitation.

^d^ TR, tricuspid valve regurgitation.

^e^ AR, aortic valve regurgitation.

^f^ AS, aortic stenosis.

^g^ MVR, mitral valve replacement.

^h^ TVR, tricuspid valve replacement.

^i^ AVR, aortic valve replacement.

^j^ Maze, surgical maze surgery of AF.

**FIGURE 1:**
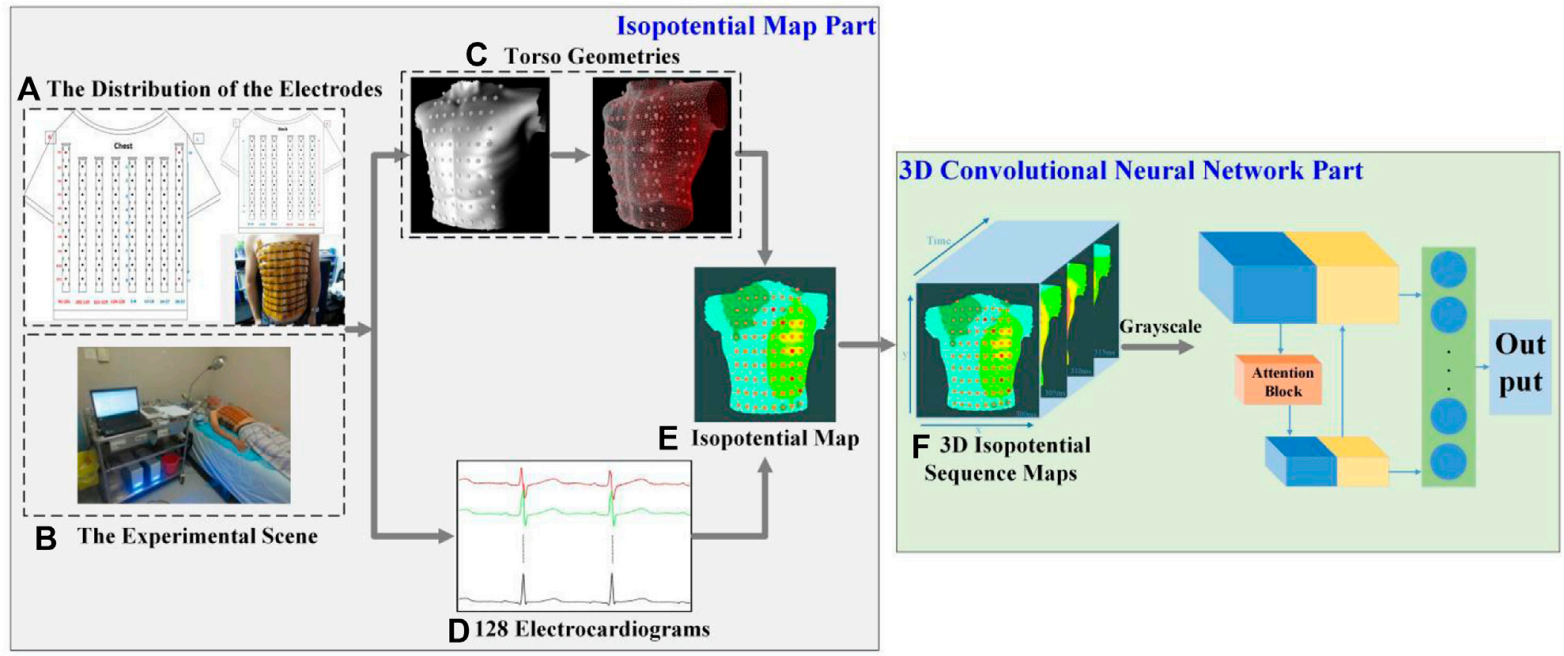
Framework of our prediction model of AF recurrence. **(A)** The distribution of the electrodes. There are 128 electrodes, including 74 on the chest and 54 on the back. **(B)** The experimental scene. **(C)** Torso geometries. Torso geometries consist of 128 body surface electrodes and a body torso geometry. **(D)** 128 electrocardiograms. Different colors show the BSPM of different channels, and there are 128 channels in total. **(E)** Isopotential map. Different colors indicate different voltage amplitudes, and the darker the color, the lower the voltage. **(F)** 3D isopotential sequence maps. y and x are the height and width of isopotential map, and time is consistent with the time of the BSPMs.

A 128-lead vest connected by elastic bands constitutes the front-end signal acquisition equipment. Every electrode is gold-plated copper, and all electrodes were gathered on a soft PCB board. Figure 1A illustrates how the electrodes were distributed on a patient’s body surface. There were 74 electrodes distributed on the anterior body surface, while 54 were distributed on the posterior body surface. Two adjacent electrodes belonging to the same column were 3.5 cm apart. At the same time, three electrode points were located in a triangular shape to construct the Wilson’s center terminal as the reference point. This reference point should be subtracted by the voltage value acquired at other points to obtain the ultimate voltage value. Data recording and storage uses the multi-channel electrophysiological signal acquisition and recording system NeuroScan (ESI-128, Compumedics Ltd., Australia) (Zhang et al., 2018). Figure 1B depicts the experimental scene.

The entire experiment was verified on the AF signals database before surgery; this is different from other work which used sinus rhythm before AF (Sahadevan et al., 2004), and where patient follow-up was conducted by the same doctor performing the same surgical procedure, which can ensure that the initial conditions of sample are the same. In this study, preoperative signals were used to predict postoperative AF recurrence, and postoperative sinus rhythm signals were used to analyze the conduction law of AF cardiac activation on BSPMs.

### 2.2 Framework overview

As shown in Figure 1, the two parts of this study were an isopotential map and a 3D-CNN. In the former, the data analyzed in this study were all BSPMs of patients with AF and were filtered by the NeuroScan system at a 1–40 Hz band-pass. After obtaining the torso geometry by pre-processing technology based on a point cloud from a laser scanning system (Guo et al., 2020), the BSPMs were interpolated to 3D displacement by inverse distance weighting (IDW). The patient’s isopotential map was displayed at the same time, and the conduction law of cardiac electrical activity was analyzed by BSPM. At the same time, the noise of baseline wander (in record “bw”) (Goldberger et al., 2000) with a signal-to-noise ratio of 12 dB was added to the original signal. In the 3D-CNN part of this study, the isopotential map was generated from the signal and transformed into 3D isopotential sequence maps by combining time information. A temporal-attention block for ECG signals was designed to predict the recurrence of AF. Figure 1F shows the 3D isopotential sequence maps. In order to process 3D isopotential sequence map information more efficiently, the original isopotential map was transformed into a gray-scale image with only one channel, whereas the color image has three (RGB). The image input to the CNN is significantly increased if a color image is employed, as it is three times larger than a gray-scale image. A more effective gray-scale image was employed because it can capture different potentials at different pixel values; this can already reflect the conduction of the ECG signal. The deep CNN is used to train the processed 3D isopotential sequence maps.

The 128-channel unipolar BSPMs at about 3 min per patient were collected, and the BSPMs were sampled at 1000 Hz. Afterward, the original signal was cut into 2 s for analysis. Segments with extremely poor signal quality were manually eliminated due to circumstances such as patient movement during the acquisition process. Consequently, the number of segments saved varies for each patient, as shown in Table 1 with specific subject information. The ratio of non-recurrent to recurrent segments is 813:359. There is a great imbalance in the amount of data. The overlap method is used to deal with recurrent samples. It should also be noted that the shift between two segments is equal to 175 points (Oliver et al., 2018; Andersen et al., 2019). Thereafter, the total data were 1627 segments, including 814 recurrent and 813 non-recurrent segments.

The development environment of this research is the Win 10 system, 64 GB memory, i7-8700 CPU, and RTX2080 GPU. The isopotential map compiler using the C++ development language adopts Visual Studio 2013, and the deep learning framework is the Tensorflow framework based on Python.

### 2.3 Isopotential map

Using the scanning platform, the 3D model of the torso geometry is reconstructed by point-cloud technology (Chen et al., 2013). The hardware is based on a Raspberry PI 3B + microcontroller, stepper motor and laser drive circuit, scanning tables, and optical sensor. Depth information is point-cloud information, which had to be collected at different sites of the torso by infrared cameras around the body. Then, the data collected in the space were processed and recovered by software, and the geometric shape of the torso geometry and the position of the surface electrodes were finally obtained. The format of the point cloud information is an obj file containing 83,184 vertices and 39,504 faces. There were a total of 129 meshes representing 128 body surface electrodes and a body torso geometry (for the latter, see Figure 1C).

IDW is a computational method based on the geometric relationship between interpolated objects (Shepard, 1968). The distance between the known point and the point to be interpolated is the “weight value”, and the interpolation points can be estimated by a weighted average. Assuming the known point is $D_{i}\left(x_{i},y_{i}\right)$ , whose value is represented by $z_{i}\left(x_{i},y_{i}\right)$ , the point to be interpolated is P(x,y) , while $d_{i}$ represents the distance between the two points P(x,y) and $D_{i}\left(x_{i},y_{i}\right)$ . The interpolation function can therefore be expressed as

$$f_{1,x}\left(x,y\right)=\frac{\sum_{i=1}^{N}\sum_{j=1,i\neq j}^{N}{\left(d_{i}\right)}^{-\left(u-2\right)}{\left(d_{j}\right)}^{-u}\left(x-x_{i}\right)z_{i}\left(z_{i}-z_{j}\right)}{{\left|\sum_{i=1}^{N}{\left(d_{i}\right)}^{-u}\right|}^{2}}$$ (1)

By replacing $\left(x-x_{i}\right)$ with $\left(y-y_{i}\right)$ , the interpolation of the $f_{1,y}\left(x,y\right)$ can be calculated. The weight of the distance is as follows:

$${\left(d_{i}\right)}^{-2}=\frac{1}{\left[{\left(x-x_{i}\right)}^{2}+{\left(y-y_{i}\right)}^{2}\right]}$$ (2)

Empirically, with the increase of the coefficient u , the interpolation points become smooth, but the computational overhead increases significantly. Usually, the parameter u is taken to be 2.

The different potential in the isopotential map was rendered as different colors filling the vertex coordinates at the same time (Abildskov et al., 1976). The 3D characterization process of the ECG data from the BSPMs is principally divided into the following steps:

1) Collect the synchronous BSPMs with a certain sampling frequency =1000 Hz , as shown in Figure 1A, according to the placement location of the acquisition electrode.
2) Remove noise or interference from power frequency, breathing or muscle power from the ECG signal collected in Step 1), and normalize the signal amplitude.
3) Based on the voltage amplitude after normalizing each of the ECG data obtained in Step 2), draw the isopotential map according to the IDW interpolation algorithm and estimate the voltage. Then map the actual or interpolated voltage values to the corresponding spatial coordinates, with different voltage values rendered into different colors according to the voltage level.
4) Repeat Step 3) to obtain the isopotential map of each sampling time by the sampling interval 1/fs and complete the dynamic rendering, then render one image at each sampling interval and save the isopotential map of each sampling time.
5) Within the period of time l , the isopotential map obtained in Step 4) is synthesized to a 3D isopotential sequence map at a fixed time interval Δt . The 3D isopotential sequence map retains the color information contained in each isopotential map rendering. Multiple series of time dimensions are merged into a 3D isopotential sequence map. The fixed time interval is Δt=1/fs , for a certain period of time l=K·Δt , where K is the number of isopotential maps included in each synthesized 3D excited sequence map. In this study, l=2 s , Δt=1 ms , K=2000 .
6) Repeat Step 5) to obtain the 3D isopotential sequence maps until all isopotential maps are traversed.

We give different colors to different voltage values according to the voltage level: the darker the color, the lower the voltage. Red represents a wave crest, and blue represents a wave trough. The research uses the OpenGL graphics interface in Visual Studio 2013 software to load the 3D torso model, obtain the isopotential map at each sampling time at a time interval of 1 ms (i.e., 1/ fs ), and use the screen capture function glReadPixels in OpenGL to save the image at each sampling time.

In our study, we use a CNN to analyze the 3D isopotential sequence maps, as too much input will increase the difficulty of network convergence. Thus, only the isopotential map from the front part of the torso is included, and the information from the back part is totally ignored.

### 2.4 Architecture and training of the prediction model

**FIGURE 2:**
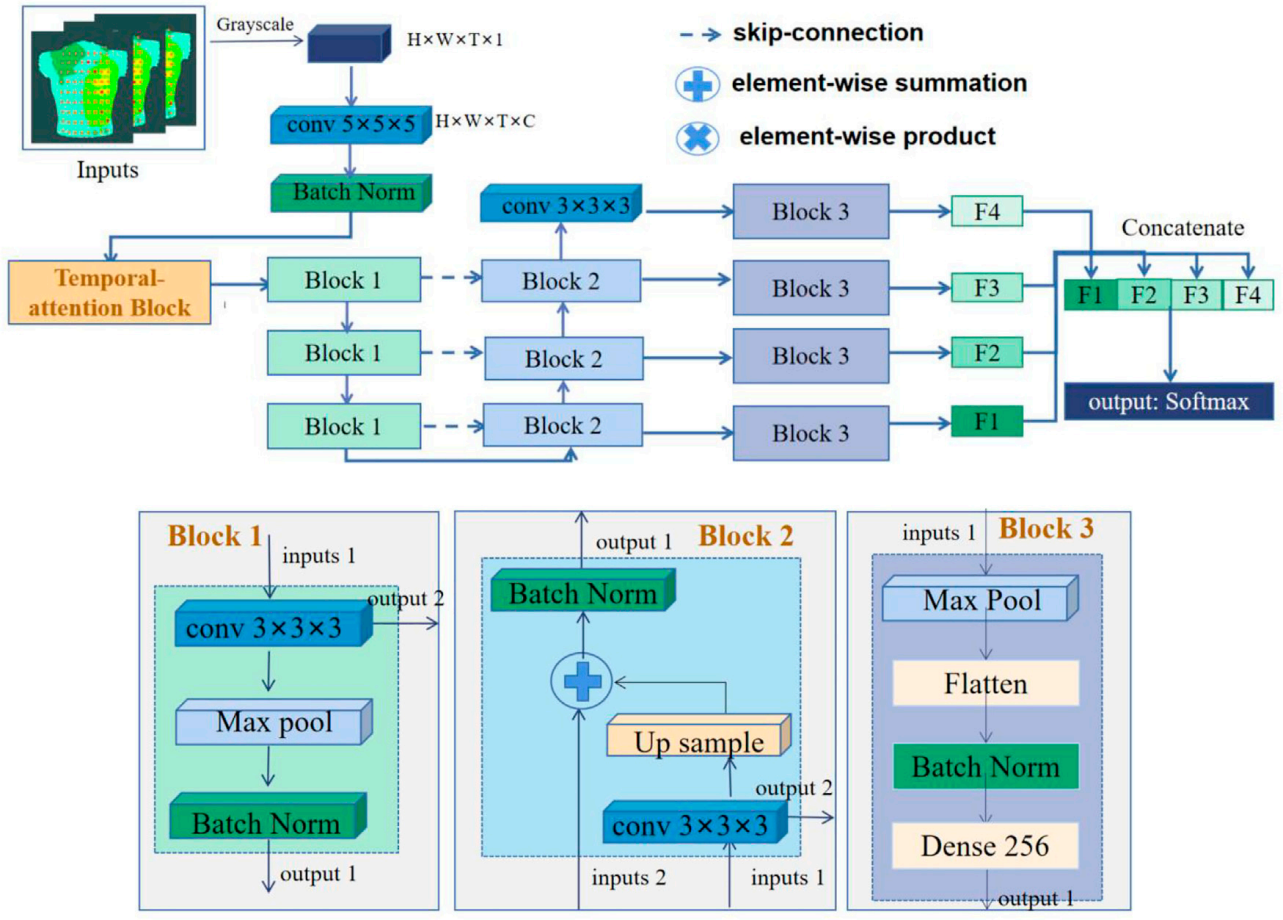
Structure diagram of the 3D-CNN classification framework.

We used 3D-CNN to predict AF recurrence. The architecture of the network is shown in Figure 2. The network takes 3D isopotential sequence maps as input and the vector representing recurrence or non-recurrence as output. The 3D isopotential sequence maps generated by a series of 2D isopotential maps as the dataset is input into the 3D-CNN. The size of the 3D isopotential sequence maps is W×H×T , where *W* indicates its width, *H* its height, and *T* is the number of frames of the 3D isopotential sequence maps. We arrive at an architecture consisting of eight convolutional layers, three fully connected layers, and a Softmax.

#### 2.4.1 3D-CNN

In order to make the optimization of such a network tractable, we employed skip connections in a similar manner to those found in the U-Net architecture (Ronneberger et al., 2015). The skip connections between neural network layers optimize training by allowing the information of low- and high-resolution features to propagate effectively in different layers of a neural network. The network architecture is illustrated in Figure 2, including Blocks 1–3 and the temporal-attention block. Block 3 is the full connection layer block. The structures of other parts consist of a contracting path as shown in Block 1 and an expansive path as shown in Block 2. The contracting path follows the typical architecture of a convolutional network. At each down-sampling step, we doubled the number of feature channels. Every step in the expansive path consists of an up-sampling of the feature map followed by a 2×2 convolution (“up-convolution”) that halves the number of feature channels. Based on the U-Net architecture, the model extracts the feature on the output of the multi-scale convolutional layer in the contraction path and inputs to the fully connected layer. The prediction result is obtained through Softmax. In Figure 2, *C* indicates the channel of the network and Dense 256 indicates that the length of the output feature vector is 256.

In the output part, the deep and shallow features of the network can be fused by fusing the information of different layers of the network. Among them, the network parameter F1 is the output after the fifth convolution layer, F2–F4 process the features after using up-convolution fusion on deep and shallow features, and the deep and shallow features are fused again through concatenating.

#### 2.4.2 Temporal attention

The temporal-attention block presented was mainly inspired by SENet in 2017 (Hu et al., 2018) and the characteristics of ECG. The prediction of AF recurrence mainly focuses on the signals within a particular time. For example, for AF recurrence, we mainly focused on the characteristics of atrial activations (Heijman et al., 2021). The data used in this study include ventricular and atrial activations. However, we paid more attention to the atrial signal for the recurrence of AF. In order to better identify the characteristics of atrial activation, we added a temporal-attention block to the network so that the signal can pay attention to K of W×H×T×C . For AF recurrence, the temporal-attention block should give greater weight to the time period of atrial activation, so that the network can pay more attention to the time period related to AF recurrence.

A temporal-attention block is a computational unit which can be built upon a transformation $F_{tr}$ mapping an input $X\in R^{H^{2032}\times W^{\prime }{\times T}^{\prime \times }C^{\prime }}$ to feature maps $U\in R^{H\times W\times T\times C}$ . In the following notation, we take $F_{tr}$ to be a convolutional operator and use $V=\left[{vs}_{1},{vs}_{2},\cdots {,vs}_{c}\right]$ to denote the learned set of filter kernels, where ${vs}_{c}$ refers to the parameters of the *c*th filter. We can then write the outputs as ${us}_{l}$ , and ${us}_{l}$ refers to the parameters of the l -th part of feature maps. That means ${vs}_{l}\in R^{a\times b\times c}$ and $X_{l}\in R^{a\times b\times c}$ , where:

$${us}_{l}={vs}_{l}*X_{l}=\overset{C}{\underset{k=1}{\sum }}{\left(\overset{i=a}{\underset{i=1}{\sum }}\overset{j=b}{\underset{j=1}{\sum }}\overset{z=c}{\underset{z=1}{\sum }}{vs}_{ijz}\times X_{ijz}\right)}_{k}$$ (3)

For a temporal feature, the traditional 3D convolution is the convolution sum of the length, width, and time dimensions of the signal. The characteristic relationship of the temporal and spatial information is thus learned by the convolution kernel, and even channel information will be mixed together through summation. The purpose of temporal attention is to extract the temporal information from this mixture so that the model can learn the temporal information more directly.

The 4D features are passed through a 1×1×1 convolution kernel, and the channel number is adjusted to 1 to obtain F through a reshape operation, where $F\in R^{H\times W\times T}$ (Szegedy et al., 2015). Since convolution is only operated in a local space, it is difficult to observe the relationship between the local and global space. Using the squeeze operation proposed by SENet, we encode all spatial features at a time into a global feature, which is generated into temporal-wise statistics by global average pooling (AvgPool) and maximum average pooling (MaxPool) (Woo et al., 2018). The temporal weight $M_{T}$ is obtained by fusing the features of global average pooling and max average pooling. $M_{T}$ goes through a sequence and excitation operation. The shared network is composed of a multi-layer perceptron (MLP) with one hidden layer. After the shared network is applied to the block, we merge the output feature vectors using element-wise summation. The formula of the temporal weight $M_{T}$ is as follows:

$$M_{T}\left(F\right)=\sigma \left(MLP\left(AvgPool\left(F\right)\right)+MLP\left(MaxPool\left(F\right)\right)\right)=\sigma \left(W_{2}ReLU\left(W_{1}\left(F_{avg}^{sq}\right)\right)+W_{2}ReLU\left(W_{1}\left(F_{\max}^{sq}\right)\right)\right)$$ (4)

where σ denotes the Sigmoid function, $W_{1}\in R^{T/r\times T}$ and $W_{2}\in R^{T\times T/r}$ . The formula of AvgPool $F_{avg}^{sq}$ and MaxPool $F_{\max}^{sq}$ are as follows:

$$F_{avg}^{sq}=\frac{1}{H\times W}\overset{H}{\underset{i=1}{\sum }}\overset{w}{\underset{j=1}{\sum }}f_{t}\left(i,j\right)$$ (5)

$$F_{\max}^{sq}=\max\left(f_{t}\left(i,j\right)\right)$$ (6)

**FIGURE 3:**
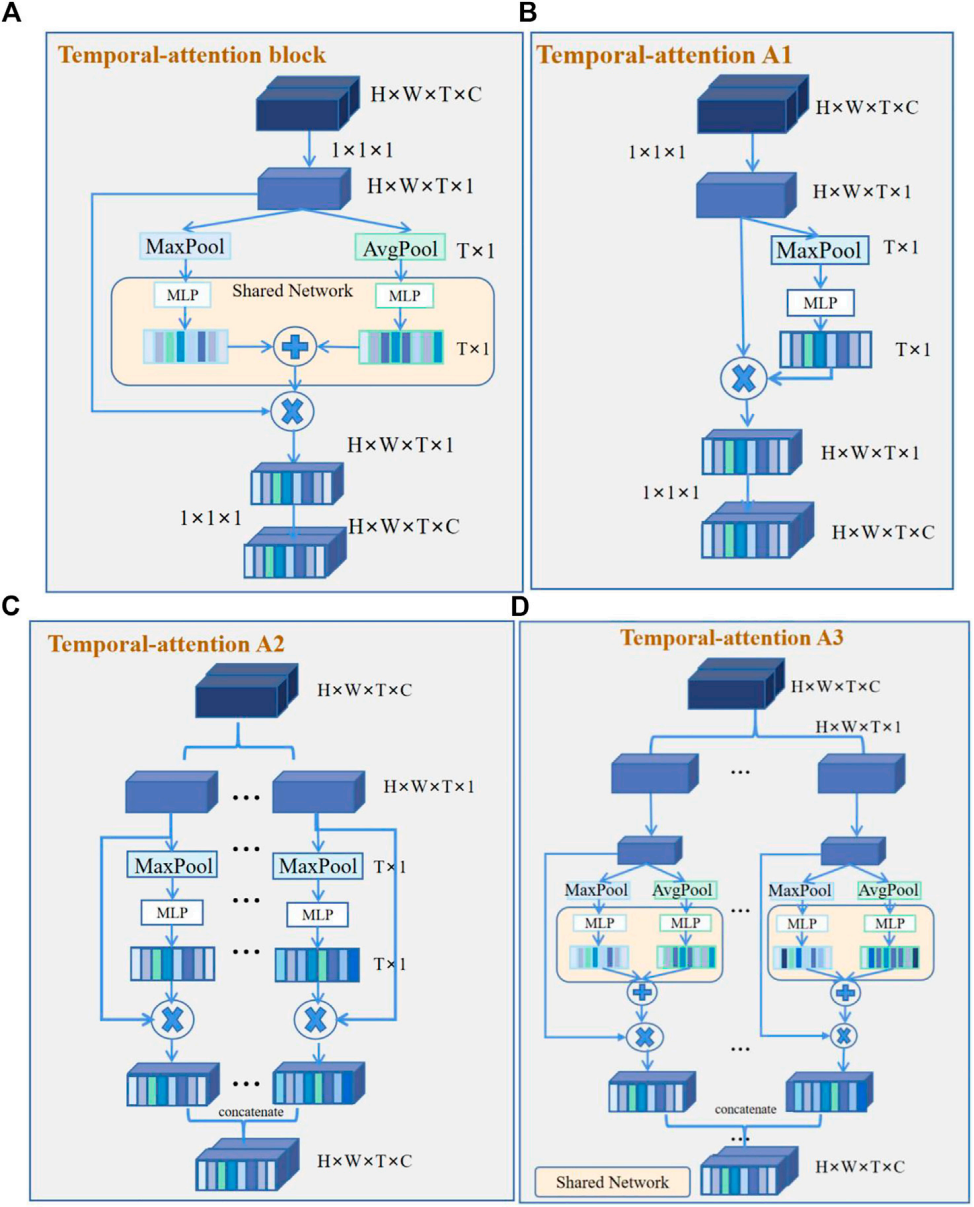
Temporal-attention block and its variants. **(A)** Temporal-attention block. Two groups of features were obtained by global average pooling (AvgPool) and maximum average pooling (MaxPool), then get the sum of the different features matrices. **(B)** Temporal-attention A1. Feature acquisition contains only MaxPool. **(C)** Temporal-attention A2. Different channel features were obtained by MaxPool, and use concatenation to fuse different channel features. **(D)** Temporal-attention A3. Different channel features were obtained by AvgPool and MaxPool, and use concatenation to fuse different channel features.

As with SENet, r is a super parameter for dimensionality reduction. In this experiment, *r* = 4 is taken. The weighted temporal attention is obtained by summing up the elements to map the features and multiplying this by the original signal *F*. Finally, the number of channels is adjusted to C using a 1×1×1 convolution kernel. The temporal-attention block structure is shown in Figure 3A, while Figures 3B–D are all variants of the structure. The temporal attention A1 structure includes a 1×1×1 convolution kernel and just uses MaxPool to generate temporal-wise statistics. Temporal attention A2 and A3 structures use concatenation to fuse different channel features instead of a 1×1×1 convolution kernel.

### 2.5 Optimization

There are more non-recurrent than recurrent samples, which causes data imbalance. Therefore, the model fails to learn the features of fewer classes and is trained with low efficiency, as most locations are easy negatives that contribute no useful learning signal. The purpose of using focal loss is to solve the serious imbalance in the proportion of non-recurrent and recurrent samples (Lin et al., 2017) and to reduce the weight of a large number of easy-to-classify samples in training. Focal loss reduces the contribution of samples which are easy to classify to loss and makes the model attend more to the hard-to-classify samples. The formula is as follows:

$${L\;}_{fl}=\left\{ \begin{array}{c} {-\alpha \left(1-y^{\prime }\;\right)}^{\gamma }\;\log\left(\;y^{\prime }\right),\;y=1\\ {-{\left(1-\alpha \right)y}^{\prime }\;}^{\gamma }\;\log\left(1-y^{\prime }\right),y=-1 \end{array}\right.$$ (7)

As aforementioned, y∈{±1} specifies the ground-truth class and $y^{\prime }\in \left[0,\;1\right]$ is the model’s estimated probability for the class with label *y* = 1, which means recurrent samples. When *γ* = 0, ${L\;}_{fl}$ is equivalent to cross entropy (CE) and as *γ* is increased, the effect of the modulating factor is likewise increased. The γ reduces the contribution of easy-to-classify samples to loss. The α can be used to balance the uneven number of non-recurrent and recurrent samples. In our study, we set γ to 2 and α to 0.25.

After each convolutional layer, we applied batch normalization (Ioffe and Szegedy, 2015) and a rectified linear activation. We also applied dropout (Srivastava et al., 2014) between the skip-connection layers and the fully-connected layers. We used the Adam (Kingma et al., 2014) optimizer with default parameters and reduced the learning rate by 1*/t* decay, where *t* denoted the training step. During optimization, we saved the best model as an evaluation of the validation set.

### 2.6 Evaluation index of performance

In our study, we used two dataset evaluation methods to test performance. One is inter-patient evaluation, which strictly requires that the training set and testing set data come from different patients (Nguyen et al., 2019). The other is intra-patient evaluation, which completely ignores the individual differences. The training set and testing set can come from the same patient to achieve higher performance. At this time, the negative impact of individual differences is the least, as is the difficulty of realization.

For the two different data-set division methods, we used four main statistical indicators to evaluate this prediction model: sensitivity (*SE*), specificity (*SP*), positive predictive value (*PPV*), and accuracy (*ACC*). These expressions are given as follows:

$$SE=\frac{TP}{\left(TP+FN\right)}\times 100\%$$ (8)

$$SP=\frac{TN}{\left(TN+FP\right)}\times 100\%$$ (9)

$$PPV=\frac{TP}{\left(TP+FP\right)}\times 100\%$$ (10)

$$ACC=\frac{\left(TP+TN\right)}{\left(TP+FN+FP+FN\right)}\times 100\%$$ (11)

where *TP* is the amount of AF recurrence samples that were correctly predicted, *TN* is the AF non-recurrence samples which were predicted as non-recurrence, *FP* indicates the AF non-recurrence samples that were wrongly predicted as recurrent, and *FN* is the recurrence samples that were wrongly predicated as non-recurrent. Another quality of the prediction model is measured by the area under curve (*AUC*) of its receiver operating characteristic (ROC) curve based on maximized *SE* and *SP* (Fawcett, 2006).

## 3 Experimental results and discussion

### 3.1 Cardiac axis

The signal of normal sinus rhythm is selected to calculate the cardiac axis. The conduction law of cardiac activations in the BSPMs is that these conduct along the direction of the cardiac axis, allowing the cardiac electrical signals of different propagation orders to be extracted through the BSPMs.

**FIGURE 4:**
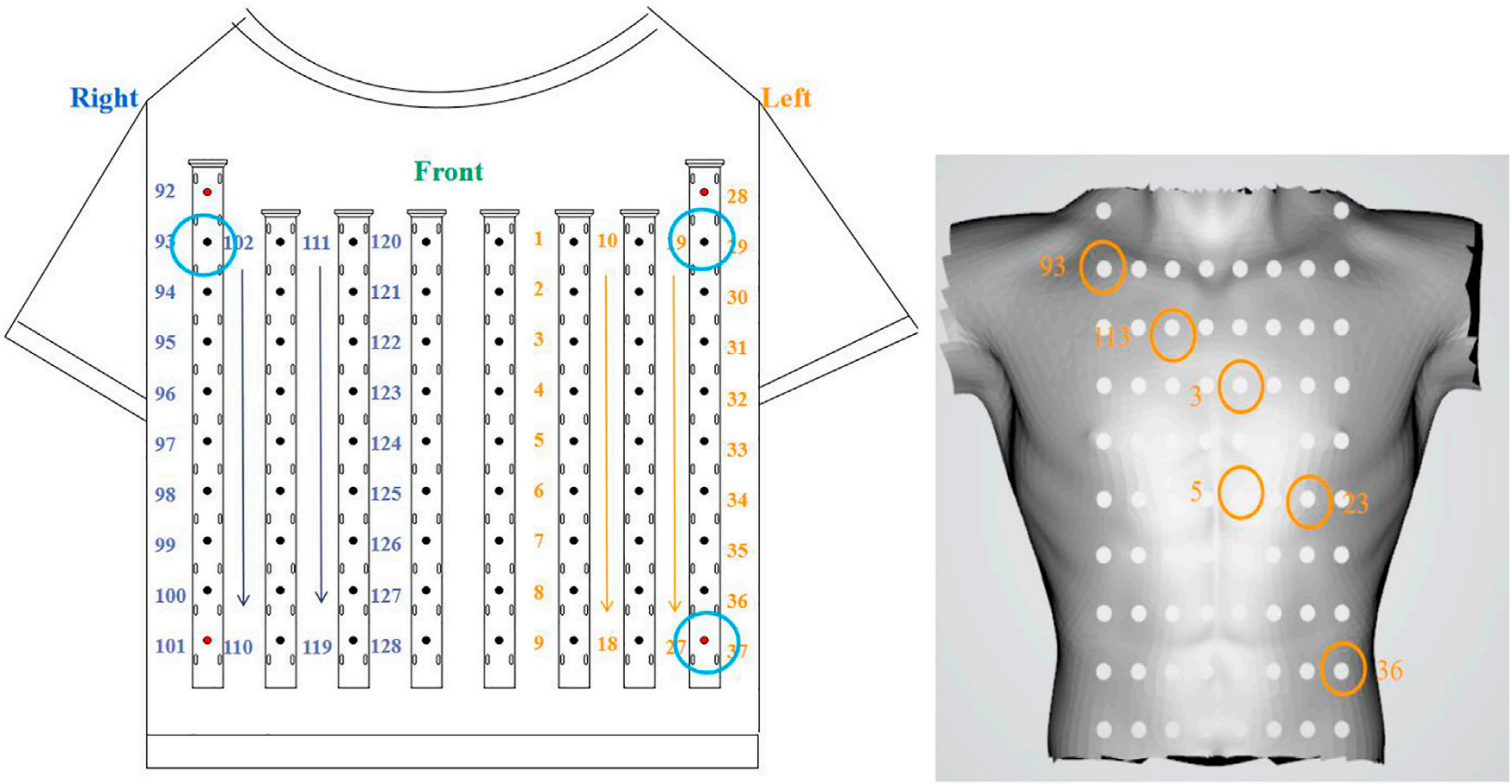
Position of six electrode reference points.

According to the electrode distribution of the anterior chest mapped on the body surface (Figure 4), we use channels 93, 29, and 37 to approximately calculate leads I and III. Lead I is approximately the difference between channels 29 and 93 and lead III is approximately the difference between channels 37 and 29. Its formula is as follows:

$$U_{Ⅰ}\;∼\;U_{29}-U_{93}$$ (12)

$$U_{Ⅲ}\;∼\;U_{37}-U_{29}$$ (13)

**FIGURE 5:**
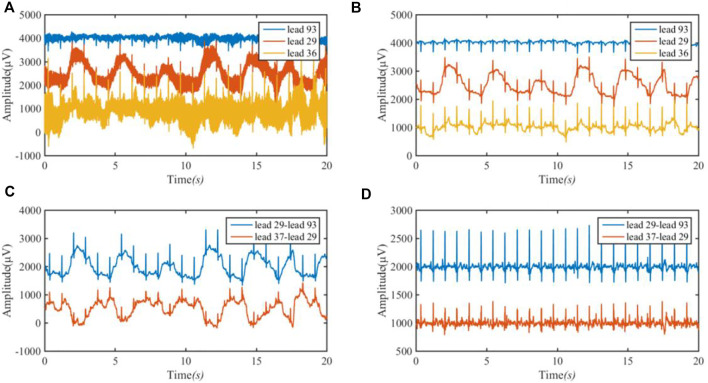
ECG of lead I (29–93) and lead III (37–29) achieved from BSPM. **(A)** the original BSPM. The blue line is 93 lead (channel); the red line is 29 lead (channel); and the orange line is 36 lead (channel). **(B)** The ECG after band-pass filtering. **(C)** The approximate ECG of lead I and III. The blue line is the approximate ECG of lead I by subtracting lead (channel) 93 amplitude from lead (channel) 29 amplitude; the red line is the approximate ECG of lead III by subtracting lead (channel) 29 amplitude from lead (channel) 37 amplitude; **(D)** The signal after removing the baseline wander.

Figure 5 shows the approximate ECG of leads I and III achieved from BSPMs. Figure 5A is the original BSPM, Figure 5B is the ECG after band-pass filtering at 1–40 Hz, Figure 5C is the approximate ECG of lead I obtained by subtracting the corresponding lead, and Figure 5D is the signal after removing the baseline wander using the low-pass filter of 3 Hz (first-order Butterworth filter).

We calculated the amplitude of positive and negative R waves in 80 s signals to obtain the patient’s cardiac axis. The sum of the amplitude of a QRS wave of $U_{Ⅰ}$ is $U_{Ⅰ}=383.7130$ and the sum of the amplitude of a QRS wave of $U_{Ⅲ}$ is $U_{Ⅲ}=176.5790$ . The cardiac axis angle is 47.9516°, which is in normal range.

**FIGURE 6:**
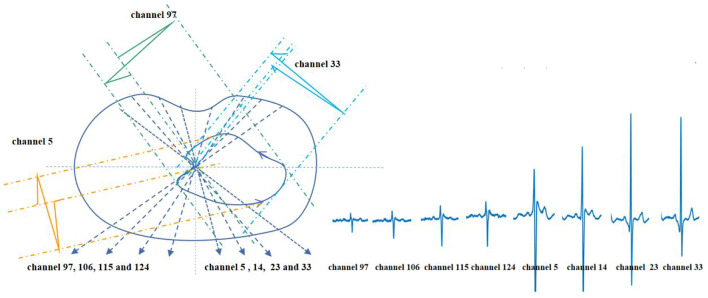
Projection and waveform formation of the transverse plane vectorcardiographic loop of BSPM.

On the transverse plane, the projection of the vectorcardiographic loop of the BSPMs is shown in Figure 6. Electrodes 97–33 in the same row of BSPMs are selected, and the propagation law of BSPMs is obtained through a two-step projection of a spatial vector cardiogram, as shown in Figure 6. By comparing the BSPMs collected in Figure 6, it is evident that the BSPMs follow the pattern of the conduction law of cardiac activations and that the peak value of the R wave follows the propagation order from left to right.

### 3.2 Verification isopotential map

For postoperative sinus rhythm, a total of 80 s sinus mapping signals (including 92 heartbeats) are included to verify the performance of the rendered dynamic mapping data. Six electrode reference points—nearly consistent with the normal electrical axis of the heart (Figure 4)—are channels 93, 112, 3, 5, 24 and 36, respectively. These channels are numbered 93 (①), 112 (②), 3 (③), 5 (④), 25 (⑤), and 36 (⑥) from small to large, and the chronological order of the QRS complex received at these six electrodes is also counted. Table 2 shows numbers and arrows being used to indicate the order of the body surface activation sequence during sinus rhythm. For example, ①→②→③→④→⑤→⑥ indicates that the activation sequence is conducted from right to left, from the top to bottom, and from channel 92 (①) to channel 36 (⑥).

**TABLE 2:** Statistics for the excitement sequence of sinus rhythm.

Activation sequence	The number of heartbeats	Delay time	The proportion of the number of heartbeats in the delay time	
①→②→③→④→⑤→⑥^a^	78	0	—	
**②**→①→③→④→⑤→⑥	11	1–3 ms	1 ms	91.67%
			3 ms	8.33%
**⑥**→①→②→③→④→⑤	2	>3 ms	100.00%	
**②**→①→**⑥**→③→④→⑤	1	1 ms	100.00%	

^a^ ①→②→③→④→⑤→⑥ indicates that activation sequence is conducted from channels 93 (①) to 36 (⑥).

The bold values represents the optimal result of different algorithms.

The delay time is used to represent the difference between the time when the electrodes with a different activation sequence receive the ECG activation and when they receive the ECG activation under normal conditions. It can be seen from Table 2 that an activation delay was detected in electrodes ② and ⑥. The longest activation delay was less than 3 ms. It is found that the delay time is short and will not have a great impact on the model rendering. Compared with the 14 times of activation delay, the difference is not obvious; this indicates that the isopotential map can approximately represent the conduction law of cardiac electrical activity on BSPMs.

**FIGURE 7:**
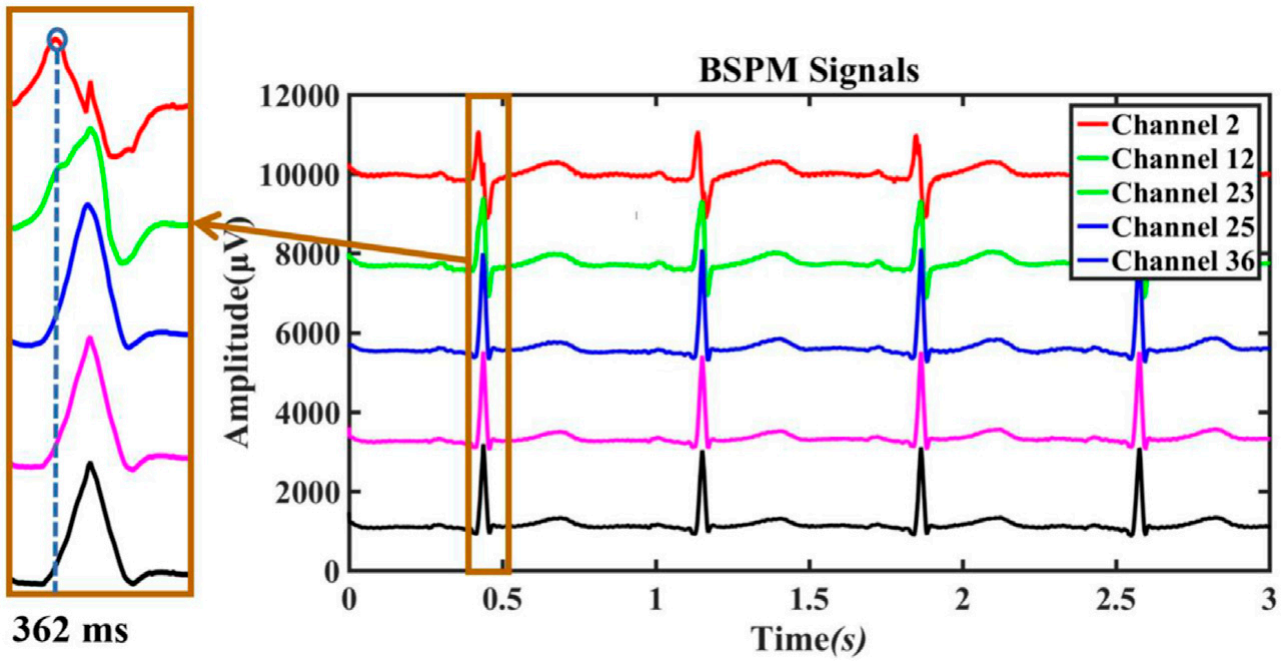
Original ECG data collected by channels 2, 12, 23, 25, and 36.

**FIGURE 8:**
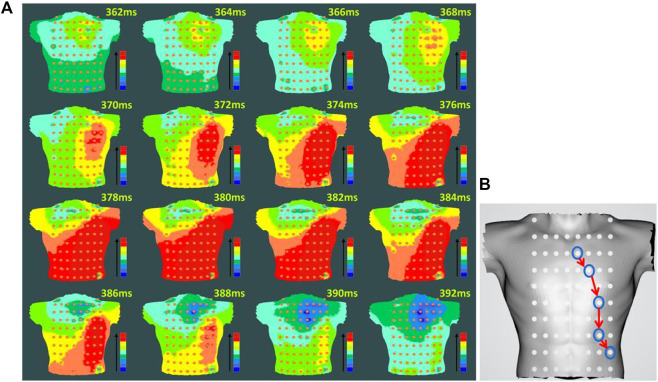
Rendering of the isopotential map of preoperative AF **(A)** Rendering of the isopotential map at different times. The time in this figure means each sampling time of the isopotential map and the color of each isopotential map represents the magnitude of the voltage amplitude: red represents the maximum relative amplitude, and blue represents the minimum. **(B)** Conduction order of Torso geometries. Blue circular lines are the location of the different electrodes; red arrow indicate the order of cardiac activations.

For preoperative AF, the BSMPs of the five selected electrode points (channels 2, 12, 23, 25, and 36) in the normal activation sequence and the rendered isopotential map are shown in Figures 7 and 8. In Figure 7, the ECG signals of the five selected channels within 3 s are arranged in parallel from top to bottom. The dotted line indicates that the time from channel 2 to the QRS complex peak is 362 ms, corresponding to the first isopotential map of Figure 8. It is evident that channel 2 first detected the moment of excitation and that the other channels also detected excitation after a certain delay—consistent with the conduction results shown in Figure 8. It can be seen from Figure 8 that the color of the place near channel 2 changes first, indicating that the excitement is first transmitted to this place. Then, along the electrical axis, the color of the lower-left area of the torso turns from yellow to red and spreads out, indicating that the excitement is transmitted to this area later.

### 3.3 3D-CNN in the intra-patient evaluation

In our study, a 3D isopotential sequence map was used to predict the recurrence of AF, and the 3D-CNN structure was used for prediction. In the meanwhile, the classification performance of four classic network structures of image was compared. Four common network training models—LeNet (Lecun et al., 1998), AlexNet (Krizhevsky et al., 2012), VGGNet-16 (Simonyan and Zisserman, 2014), and ResNet (He et al., 2016)—were selected for comparison with the 3D-CNN classification model in this study. The input of LeNet, AlexNet, and VGGNet-16 was 3D isopotential sequence maps changed from a traditional 2D image; ResNet was changed from ResNet 50 and had 16 convolution layers and 1 max average pooling layer.

In our experiment, different sizes of 3D isopotential sequence maps were reserved to compare the prediction results. Because the data size is too large when *K* = 2000, in order to save training time and improve network performance, the network input size *W* × *H* × *T* is 32 × 32 × 128, 64 × 64 × 256, and 64 × 64 × 400, respectively. We randomly divided the dataset (training set: validation set: testing set = 7: 2: 1). The results of the comparison of five different 3D network models and four different input sizes are shown in Table 3.

**TABLE 3:** Comparison of balanced random prediction performance with different network structures.

Size	Model	PPV(%)	SP(%)	SE (%)	ACC(%)	Time s/epoch	Batch size
32 × 32×128	LeNet	96.34	96.29	97.53	96.91	5 s/epoch	16
	AlexNet	65.85	48.14	100.00	74.07	11 s/epoch	16
	VGGNet-16	100.00	100.00	93.82	96.91	26 s/epoch	16
	ResNet	100.00	100.00	97.53	98.76	3 s/epoch	16
	**Proposed**	98.77	100.00	100.00	**99.38**	8 s/epoch	16
64 × 64 × 256	LeNet	92.11	92.59	86.42	89.51	8 s/epoch	16
	AlexNet	OOM^a^					16
	VGGNet-16	OOM^a^					16
	ResNet	97.18	97.53	85.19	91.36	19 s/epoch	16
	**Proposed**	100.00	100.00	96.30	**98.14**	52 s/epoch	16
64 × 64 × 400	LeNet	98.77	98.76	98.77	98.77	14 s/epoch	8
	AlexNet	OOM^a^					8
	VGGNet-16	OOM^a^					8
	ResNet	100.00	100.00	97.53	98.76	31 s/epoch	8
	**Proposed**	100.00	100.00	98.77	**99.38**	85 s/epoch	8

^a^ Out of memory.

The bold values represents the optimal result of different algorithms.

It can be seen from Table 2 that the performance of different models for data differs. Compared with other models, AlexNet and VGGNet-16 have insufficient memory when the input size of the model is larger than 64 × 64 × 256. This is because the model parameters are too large. From the results in the table, we can see that the training speed of the 32 × 32 × 128 model is obviously faster than that of other sizes. For the other three networks, the performance of LeNet and ResNet is unstable, while the result of 3D-CNN is the best and is relatively stable.

### 3.4 3D-CNN in the inter-patient evaluation

A training set and testing set can be derived from the same patient, so the accuracy of using neural networks to predict recurrence is very close. In order to better verify that the network proposed in this study can effectively distinguish spatial–temporal features, in the later experiments we used the inter-patient method: the method of distinguishing patients to verify the model. The experiment uses five-fold cross-validation to characterize the experimental results. Since there are only four recurrent patients, one was randomly selected for training.

The 3D 32 × 32 × 128 isopotential sequence map was selected as the input to the network, and LeNet, ResNet, and 3D-CNN were selected for comparison. Table 4 shows that, in the case of a small amount of data, the accuracy of inter-patient in predicting the recurrence of AF has reached 81.48%. It can be seen from Table 3 that the 3D-CNN performs better than the three classic image network structures with an SE of 67.71%, SP of 95.69%, and PPV of 76.79%, based on the same dataset in inter-patient prediction of AF recurrence.

**TABLE 4:** Inter-patient prediction performance of the five-fold cross-validation model.

Model	Indicator	Fold 1	Fold 2	Fold 3	Fold 4	Fold 5	Average
LeNet	ACC(%)	51.85	43.30	82.02	70.30	76.27	64.75
	SE (%)	0.00	11.79	79.49	63.31	71.43	45.20
	SP(%)	100.00	97.89	86.34	76.22	83.34	88.56
	PPV(%)	0.00	90.62	90.79	69.29	87.05	67.55
ResNet	ACC(%)	51.85	36.60	37.10	93.39	39.47	51.68
	SE (%)	0.00	0.00	0.00	94.24	0.00	18.85
	SP(%)	100.00	100.00	100.00	92.68	100.00	**98.52**
	PPV(%)	0.00	0.00	0.00	91.61	0.00	18.32
Proposed	ACC(%)	51.85	91.75	95.16	95.71	72.94	**81.48**
	SE (%)	0.00	91.87	95.97	90.65	60.07	**67.71**
	SP(%)	100.00	91.55	93.79	100.00	95.69	95.69
	PPV(%)	0.00	94.96	96.32	100	92.66	**76.79**

The bold values represents the optimal result of different algorithms.

### 3.5 Effectiveness of temporal-attention block in the inter-patient evaluation

To verify the effectiveness of the proposed components of our model, we conducted control experiments with fine-tuned models on the inter-patient dataset using five-fold cross-validation. In the control experiment, we selected 64 × 64 × 256 as the input size. The baseline represents the CNN architecture using VGGNet-5. The 3DCNN + F4 represents the proposed model with up-convolution. The results of the control experiment are shown in Table 5; the proposed 3DCNN + F4 model outperforms the traditional VGGNet structure. It can also be seen that up-convolution has excellent performance on the inter-patient dataset, which demonstrates that up-convolution can effectively expand the difference between the recurrence and non-recurrence samples.

**TABLE 5:** Performance of up-convolution model on five-fold cross-validation.

Model	PPV	SP	SE	ACC	AUC
VGG-5	**69.82**	87.08	56.66	71.48	**0.7958**
VGG-8	49.79	**96.65**	11.30	54.27	0.5370
3D-CNN + F4	64.98	82.66	**63.27**	**73.09**	0.7634

The bold values represents the optimal result of different algorithms.

To more intuitively show the advantages of fusing the deep and shallow features model in the full connection layer, we calculated the performance of the validation set a on five-fold cross-validation. As shown in Table 6, only adding the fully connected layer of F2 or F3 could not improve the network identification accuracy of recurrent AF. We speculate that F2 or F3 might contain limited information in the middle layer of the network, so it could not bring gain to the network. However, when F1 and F4 were concatenated, the model contained the fusing deep and shallow features and performed better. Furthermore, when F1–F4 were concatenated, the model contained features of different depth and achieved best performance. The focal loss is widely used in class-imbalanced classification; in our work, the default-loss function is set to focal loss in a structure containing the F1–F4 methods. Overall, these results indicate that the network model combined with features of different depths can perform better.

**TABLE 6:** Performance of fusing the deep and shallow features model on five-fold cross-validation.

Model	PPV(%)	SP(%)	SE (%)	ACC(%)	AUC
3D-CNN + F1+F4	70.37	86.96	60.26	74.19	0.8214
3D-CNN + F4+F2	67.83	**88.56**	43.58	63.74	0.7757
3D-CNN + F4+F3	67.21	81.37	54.76	67.30	0.7385
3D-CNN + F4+F1+F2+F3	**72.48**	71.59	62.10	74.81	0.8459
3D-CNN + F4+F1+F2+F3+FOCAL	71.49	85.36	**65.80**	**76.36**	**0.8766**

The bold values represents the optimal result of different algorithms.

In addition, as shown in Table 7, our experiment analyzed the network with temporal-attention structure. By comparing the attention structures of temporal-attention A1 and other structures, it is found that the 1 × 1 × 1 structure can bring gain to the network. We can see that the network model combined with temporal-attention A1 or temporal-attention block can achieve better results. It can also be seen that temporal-attention block (proposed) by fusing the features of global average pooling and max average pooling can effectively expand the difference between the recurrence samples and non-recurrence samples. Temporal-attention A3 is the most complex attention block in our experiment, while the results are not satisfactory. According to the results for temporal-attention A2 and A3, it seems that the attention-block parameters need not be too complex; otherwise, difficulties in network training will result.

**TABLE 7:** Performance of adding attention block model on five-fold cross-validation.

Model	PPV(%)	SP(%)	SE (%)	ACC(%)	AUC
Temporal-attention A1	69.86	68.02	**70.67**	73.24	0.7583
Temporal-attention A2	68.09	80.07	63.70	72.92	0.7798
Temporal-attention A3	65.05	82.41	51.53	66.26	0.7041
Temporal-attention block (Proposed)	**76.79**	**95.69**	67.71	**81.48**	**0.8850**

The bold values represents the optimal result of different algorithms.

## 4 Discussion

### 4.1 Isopotential map and its clinical significance

Many clinical indicators have been proposed to measure the recurrence of AF, such as CAAP-AF score (Winkle et al., 2016), while there is still a lack of a standard to evaluate the recurrence of AF by preoperative ECG. In this study, a new method based on 3D isopotential sequence maps is proposed to non-invasively evaluate the complex cardiac electrical activity of AF before CA.

The isopotential map shows the difference of the potential distribution of body surface ECG activity, which is a direct manifestation of the ECG conduction pathway. Over time, a series of isopotential maps on the torso geometry form the fluctuation map that represents the conduction path of cardiac electrical activity in the torso across the body surface. The experimental results show that fluctuations in the isopotential map can reveal some regularities of the conduction of the cardiac electrical activity. The 3D-CNN model could extract features of 3D isopotential sequence maps through the convolution layer. As an isopotential map is rich in spatial and temporal information, 3D-CNN can combine the spatial–temporal information using the unique skip-connections. Through the convolution layer, the detailed features reflecting the conduction of cardiac electrical activity in the isopotential map can be extracted to accurately predict the recurrence of AF.

### 4.2 Comparison with other studies

Based on the same dataset in the intra patient evaluation, the 3D-CNN performed better than the CNN approach of amplitude of discrete ECG signal, with SE of 83.50% and SP of 95.99% in predicting AF recurrence (Li et al., 2018), with SE of the proposed approach increasing by almost 15%.

Due to the lack of a public database for the study of AF recurrence, we can only make comparison with research in different datasets. Compared with the traditional approach of the P wave signal-averaged ECG method (Aytemir et al., 1999) with SE of 70% and SP of 76%, and based on the different dataset in the inter-patient evaluation, our model can achieve better prediction results by inputting 3D isopotential sequence maps that combine temporal information and spatial characteristics. Our method associates the higher spatial-temporal characteristics complexity of BSPM with successful CA procedures, even though the interclass has statistically significant differences which are not verified on the signals we examined.

### 4.3 Benefits of the classification method

Experiments show that up-convolution and skip connections can promote the network compared with the traditional VGG network. The skip connections between the neural network layers of the dense layer can also make the network integrate features of different depth and can improve the accuracy of the network in identifying the recurrence of AF. Focal loss makes the model attend better to difficult samples and can solve the problem of data imbalance, thus improving the accuracy of identifying the recurrence of AF.

Our research proposes a novel attention block—temporal attention—which captures the importance of features of the local space of ECG signals in a period of time. Temporal attention uses an efficient attention-computation method that does not have any information bottlenecks. By comparing other attention blocks, we find that, for long time-series data, the temporal-attention block we propose can effectively extract temporal information and improve the accuracy of prediction. Our experiments demonstrate that temporal attention improves the baseline performance of architectures like 3D-CNN on tasks like ECG classification or other physiological signals, while only introducing a minimal computational overhead. We suggest that this temporal-attention block can achieve good results for any type of time series.

### 4.4 General remarks and limitation

Our experiment included 14 patients in the intra-patient evaluation. We used random shuffling to choose partial segments of 14 patients to build the network model and another to test it. This method ignores patient-specific differences because training segments and test segments are probably from the same patient—leading to relatively decent results—while other patients not involved in the network model training (non-participants) will have very poor test outcomes. In order to avoid this situation, this study used the inter-patient evaluation method, where the segments participating in the network training and the tested segments come from different patients, thus avoiding the aforementioned situation.

The lack of comparison with endocardial recording has hampered our research. A global overview of cardiac electrical activity is provided by BSPMs, while endocardial signals account for local information. Nevertheless, we propose a noninvasive analysis method. The superiority of our method over conventional CA outcome predictors has been demonstrated. Furthermore, the conclusion of this study is based on the BSPMs of 1627 segments from 14 patients with AF, and there is no available public database in regard to postoperative detailed information for patients with AF. For further research, we need to gradually collect more clinical BSPM data of AF patients to further verify the reliability of the proposed methods.

## 5 Conclusion

BSPMs combined with 3D isopotential sequence maps can be used as a tool for the clinical diagnosis and treatment of AF. Isopotential maps can express the conduction law of cardiac electrical activity on the body surface. Furthermore, 3D isopotential sequence maps can obtain the spatial information of conduction. Temporal-attention block is easy to use, can be embedded in any layer of the network, and has fewer parameters. The 3D-CNN with temporal-attention block can extract the features of 3D isopotential sequence maps, and the network is shown to be robust. The optimal network combination confirmed its excellent intra-patient prediction performance with 99.38% of ACC, 98.77% of SE, 100.00% of SP, and 100.00% of PPV. In intra-patient evaluation, 3D-CNN achieved 81.48% of ACC, 67.71% of SE, 76.79% of SP, 95.69% of PPV, and 0.8850 of AUC. A 3D-CNN with temporal-attention block can provide relevant insights for selecting patients with low recurrence risk and suitability for surgery for radiofrequency ablation, thus providing better treatment for them.

## Data Availability

The original contributions presented in the study are included in the article/supplementary material; further inquiries can be directed to the corresponding author.
